# Blocking EZH2 methylation transferase activity by GSK126 decreases stem cell-like myeloma cells

**DOI:** 10.18632/oncotarget.13773

**Published:** 2016-12-02

**Authors:** Delong Zeng, Maoxing Liu, Jingxuan Pan

**Affiliations:** ^1^ Jinan University Institute of Tumor Pharmacology, College of Pharmacy, Jinan University, Guangzhou, China

**Keywords:** EZH2 inhibitor, GSK126, multiple myeloma, apoptosis, cancer stem cells

## Abstract

EZH2 is a critical epigenetic regulator that is deregulated in various types of cancers including multiple myeloma (MM). In the present study, we hypothesized that targeting EZH2 might induce apoptosis in myeloma cells including stem cell-like cells (CSCs). We investigated the effect of EZH2 inhibition on MM cells using a potent inhibitor (GSK126). The results showed that GSK126 effectively abrogated the methylated histone 3 (H3K27me3) level in MM.1S and LP1 cells, and inhibited the number of live cells and colony formation in soft agar of six MM cell lines. GSK126 induced massive apoptosis in MM.1S, LP1 and RPMI8226 cells. Progressive release of mitochondrial cytochrome c and AIF into the cytosol was detected in GSK126-treated MM cells. GSK126 treatment elicited caspase-3-dependent MCL-1 cleavage with accumulation of proapoptotic truncated MCL-1. These results suggested that GSK126 triggers the intrinsic mitochondrial apoptosis pathway. Enhanced apoptosis was observed in the combination of GSK126 with bortezomib. Using ALDH and side population (SP) assays to characterize CSCs, we found that GSK126 eliminated the stem-like myeloma cells by blocking the Wnt/β-catenin pathway. The *in vivo* anti-tumor effect of GSK126 was confirmed by using RPMI8226 cells in a xenograft mouse model. In conclusion, our findings suggest that EZH2 inactivation by GSK126 is effective in killing MM cells and CSCs as a single agent or in combination with bortezomib. Clinical trial of GSK126 in patients with MM may be warranted.

## INTRODUCTION

Multiple myeloma (MM) is a hematological malignancy that originates from long-lived terminally differentiated B cells or plasma cells (PCs) in lymph nodes which are usually then colonized in bone marrow (BM) during the disease progression. In USA and Europe, MM is the second most frequent hematological malignancy with an incidence of six per 100, 000 per year [1]. Although significant progress has been made in the treatment of MM in recent years, increasing the median survival from 3 years to 6 years [1], the disease relapse ultimately occurs in most MM patients [2, 3], who display losing response to clinically available agents. The failure of current therapies indicates the existence of a subset of drug-resistant cells which survives during the treatment and replenishes the tumor. Cancer stem-like cells (CSCs) have been suggested in various types of cancers including MM, to be the subset of tumor cells that exhibits features of drug-resistance, self-renewal and tumor-initiating. CD138^-^ [4] or CD19^-^CD45^low/-^CD38^high^CD138^+^ [5] subpopulations were reported to define stem-like myeloma cells, although it is still controversial (see review in ref. [6]). Therefore, targeting CSCs in MM, in addition to bulk tumor cells, may represent a promising strategy for MM therapy.

The stemness of CSCs is regulated by epigenetic mechanism. Class III HDAC SIRT1 controls CSCs in chronic myeloid leukemia [7]. SIRT1 inhibition induces elimination of CSCs in chronic myeloid leukemia [7]. Additionally, HDAC inhibition by pan-HDAC inhibitor SAHA also effectively kills CSCs in chronic myeloid leukemia [8]. Histone methylation is another critical factor of CSCs. Trimethylated histone H3 at lysine residue 27 (H3K27me3) is a transcriptionally repressive mark of a wide range of genes that play important roles in regulating embryonic development [9], stem cell self-renewal [10, 11] and cancer development [12]. The polycomb repressive complex 2 (PRC2) is a conserved and essential chromatin modifier that specifically catalyzes the methylation of histone H3 at lysine residue 27 (H3K27) by its catalytic subunit enhancer of zeste homolog 2 (EZH2) [13]. Overexpression or mutations of EZH2 have been reported in various types of solid and hematopoietic cancers [14–16]. In MM, EZH2 expression is up-regulated and significantly correlative with tumor burden and other important variables that historically indicates poor prognosis [17].

EZH2 inhibition or depletion has been reported to be effective in killing different types of cancer, and several selective inhibitors have been designed and investigated (see reviews in refs. [18, 19]). Three of these inhibitors [i.e., GSK2816126 (GSK126), EPZ-6438 (E7438, tazemetostat) and CPI-1205] are now undergoing phase I or II clinical trials (http://clinicaltrials.gov). GSK126, an S-adenosylmethionine (SAM) competitor, is a highly selective EZH2 inhibitor with a *K*i value of ~0.5 nM which is > 150 folds smaller than that for EZH1 [20]. Supported by *in vitro* data [20], GSK126 is now being tested in phase I clinical trial for relapsed/refractory diffuse large B cell lymphoma, transformed follicular lymphoma, other non-Hodgkin's lymphomas, solid tumors and multiple myeloma (NCT02082977, https://clinicaltrials.gov/). Although the anti-proliferation activity is intensively investigated, little is known about the pro-apoptotic effect of EZH2 inhibition on MM CSCs.

In the present study, we hypothesized that EZH2 inhibition induced apoptosis in bulk tumor cells and CSCs in MM. We tested this hypothesis by determining the anti-MM activity against MM *in vitro* and *in vivo*.

## RESULTS

### Blocking the cellular EZH2 methyltransferase activity by GSK126 counteracts growth of MM cells

**Figure 1:**
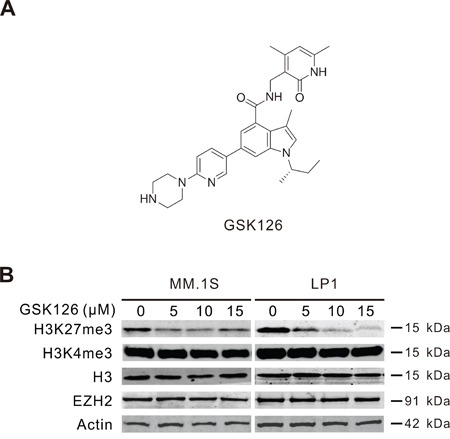
GSK126 inhibits cellular EZH2 methyltransferase activity in multiple myeloma (MM) cells **A**. Chemical structure of GSK126. **B**. MM.1S and LP1 cells were treated with increasing concentrations of GSK126 for 72 h, the whole cell lysates were subjected to immunoblotting for analysis of EZH2, H3K27me3, H3K4me3 and H3.

EZH2 expression has been reported to be absent in normal BM plasma cells, whereas it was increased in MM cells and correlative with tumor burden during the disease progression [17, 21], hinting a role of EZH2 during the tumorigenesis and progression of MM. We therefore investigated the effect of blocking the cellular methyltransferase activity of EZH2 using its specific inhibitor GSK126 (Figure 1A) on MM cells. MM.1S and LP1 cells were treated with GSK126 for 72 h, Immunoblotting results showed that GSK126 concentration-dependently decreased the levels of EZH2 specific methylation marker H3K27me3, without changes in the levels of H3K4me3, total histone H3 and EZH2 (Figure 1B).

**Figure 2:**
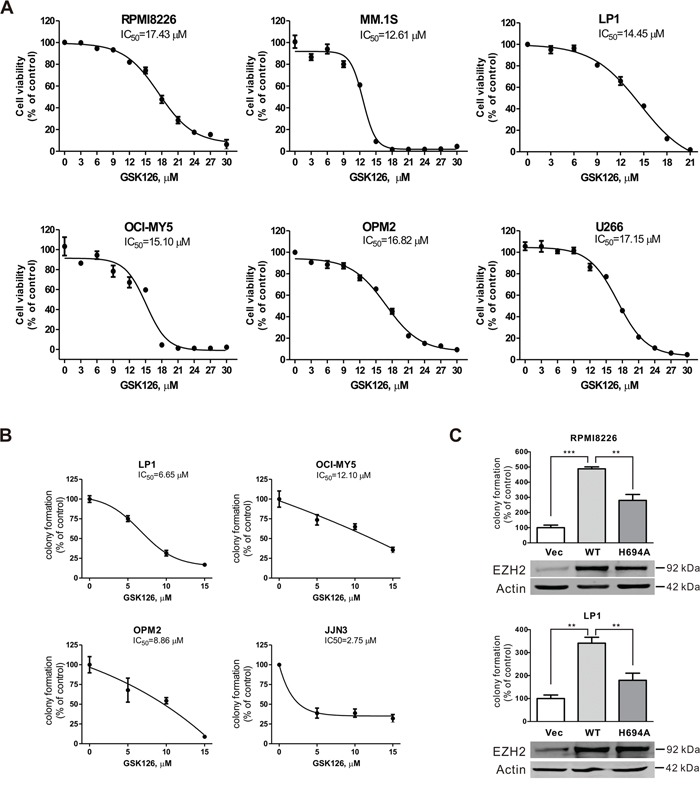
GSK126 inhibits growth of multiple myeloma cells **A**. MM cells were incubated with increasing concentrations of GSK126 for 72 h. The cell viability was measured by MTS assay. Dose-response curves are shown. **B**. Clonogenicity of MM cells were evaluated with drug-free soft agar assay in the indicated lines of MM cells after 24 h of pre-treatment with the escalating concentrations of GSK126. Curves are plotted with mean ± SEM. **C**. The colony-formation ability of RPMI8226 and LP1 cells that were transfected with constructs empty vector, EZH2-WT, or EZH2-H694A was measured in soft-agar assay, and the overexpression of wild type and mutant EZH2 was examined by immunoblotting. **, *P* < 0.01; ***, *P* < 0.0001, one-way ANOVA with *post hoc* intergroup comparison by the Tukey's test.

We next examined the effect of GSK126 on growth of MM cells. The cell viability assayed in 6 lines of MM cells exposed to escalating concentrations of GSK126 for 72 h revealed that GSK126 potently inhibited the growth of all tested cell lines, with IC_50_ values ranging from 12.6 μM to 17.4 μM (Figure 2A), suggesting that the methyltransferase activity of EZH2 may be required for the viability of MM cells and that blocking EZH2 suppressed their viability. We further investigated the effect of GSK126 on MM growth in terms of the anchorage-independent colony-formation in soft agar. The results showed that after exposure to GSK126 for 24 h, the colony-formation ability of MM cells were significantly reduced with IC_50_ < 10 μM (Figure 2B). To define the specific effect of blocking EZH2 methyltransferase activity by GSK126 on cancerous cell growth, we compared the aggressiveness of MM cells ectopically expressing constructs encoding HMT activity-dead H694A mutant and WT EZH2. As illustrated in Figure 2C, in comparison with the RPMI8226 and LP1 cells transfected with empty vector, the cells transfected with construct EZH2-WT showed an increased colony formation ability. By contrast, the cells transfected with construct EZH2-H694A displayed an alleviated clonogenicity. The results suggest that EZH2 may impact on growth of MM cells in a methyltransferase activity-dependent and -independent manner.

Taken together, these results suggested that methyltransferase activity of EZH2 is required for the growth of MM cells, and blocking the enzymatic activity by GSK126 was sufficient to repress the growth of MM cells.

### GSK126 induces apoptosis in MM cells through mitochondrial pathway

**Figure 3:**
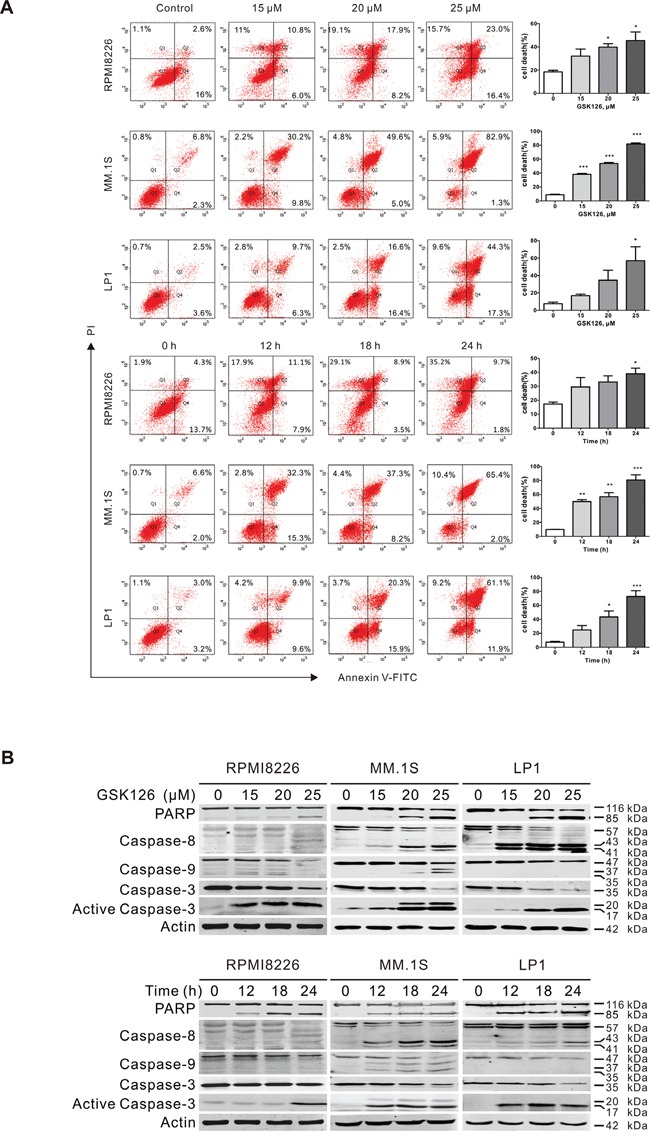
GSK126 induces apoptosis in multiple myeloma cells **A**. RPMI8226, MM.1S and LP1 cells were exposed to increasing concentrations of GSK126 for 24 h, or to 25 μM GSK126 for different time, and the apoptotic cells were analyzed by flow cytometry after dual-staining with Annexin V and propidium iodide (PI). *Left*, representative flow cytometry dot plots; *Right*, statistical analysis of 3 independent experiments. Dead cells were the sum of cells with single- or dual-stained by Annexin V or PI. *, *P* < 0.05; **, *P* < 0.01; ***, *P* < 0.0001, one-way ANOVA with *post hoc* intergroup comparison by the Tukey's test. **B**. Immunoblotting analysis was conducted for PARP, Caspase-8, -9 -3 and active-caspase-3 in RPMI8226, MM.1S and LP1 cells treated with escalating concentrations of GSK126 for 24 h, or with 25 μM GSK126 for the indicated time.

To evaluate the anti-survival effect of EZH2 inhibition by GSK126, RPMI8226, MM.1S and LP1 cells were treated with GSK126 at different concentrations or a fixed concentration for varying time, and apoptosis of the cells were analyzed by flow cytometry. The results revealed that GSK126 induced robust apoptosis in RPMI8226, MM.1S and LP1 cells in a dose- and time-dependent manner (Figure 3A). The apoptosis-indicative cleavage of PARP, caspase-8, -9 and -3 detected by immunoblotting further supported occurrence of apoptosis in RPMI8226, MM.1S and LP1 cells (Figure 3B).

**Figure 4:**
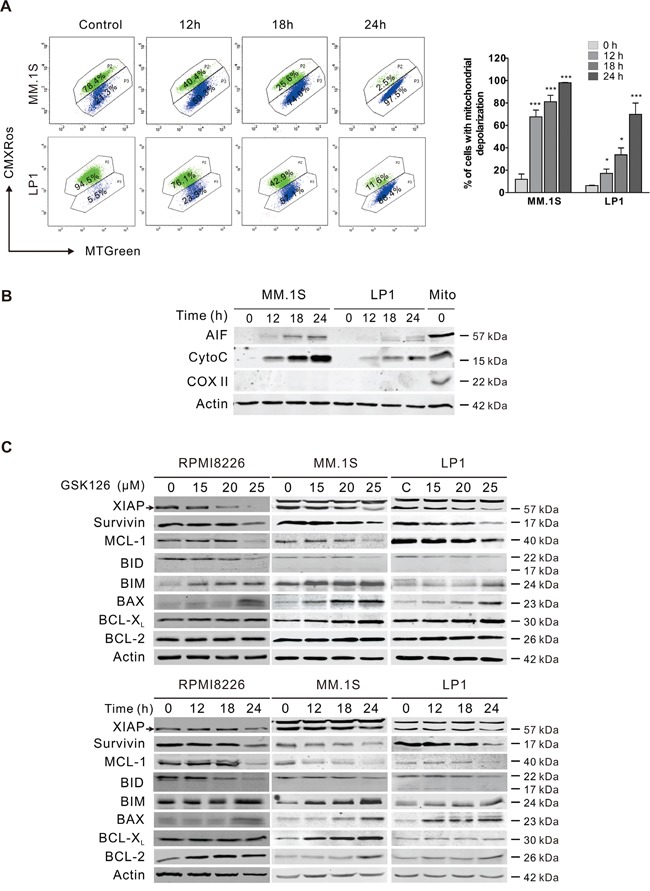
GSK126 triggers the mitochondrial pathway of apoptosis **A**. MM.1S and LP1 cells were treated with 25 μM GSK126 for the time indicated, and the mitochondrial potential was then analyzed by flow cytometry after staining with CMXRos and MTGreen. Representative dot plots (left) and statistical analyses of 3 independent experiments (right) were shown. **B**. MM.1S and LP1 cells were treated with 25 μM GSK126 for the indicated durations before the cytosolic fractions were extracted with digitonin buffer. AIF and cytochrome c (Cyto C) in the cytosol fractionations were detected by immunoblotting. Cytochrome c oxidase subunit II (COX II) served an indicator of mitochondrial extracts (Mito). **C**. Dose- and time-dependent effects of GSK126 on apoptosis-related proteins in RPMI8226, MM.1S and LP1 cells were detected by immunoblotting. Arrows indicates the specific bands of corresponding proteins.

Because the activation of caspase-9 and -3 suggested mitochondria damage, we further assessed whether there was alteration in mitochondrial transmembrane potential. After MM.1S and LP1 cells were exposed to GSK126, intracellular mitochondrial transmembrane potential detected by flow cytometry analysis based on CMXRos and MTGreen dual probing showed a marked decline (Figure 4A). The increased mitochondrial outer membrane permeabilization (MOMP) was further confirmed by the release of apoptosis-inducing factor (AIF) and cytochrome c from mitochondrial intermembrane space to the cytosol (Figure 4B). Together, the results revealed that GSK126 might trigger apoptosis in MM cells through mitochondrial pathway.

### Cleavage of MCL-1 is critical for GSK126-induced apoptosis in MM cells

To further investigate the molecular mechanism leading to apoptosis, we determined the changes of members of BCL-2 family and IAP family, which regulate the MOMP and activity of caspases. Treating RPMI8226, MM.1S and LP1 cells with increasing concentrations of GSK126 or with constant concentration for different time, resulted in a robust downregulation of XIAP, survivin, MCL-1 and Bid, and upregulation of BIM and BAX in a dose- and time-dependent manner, while BCL-X_L_ and BCL-2 were unchanged (Figure 4C).

**Figure 5:**
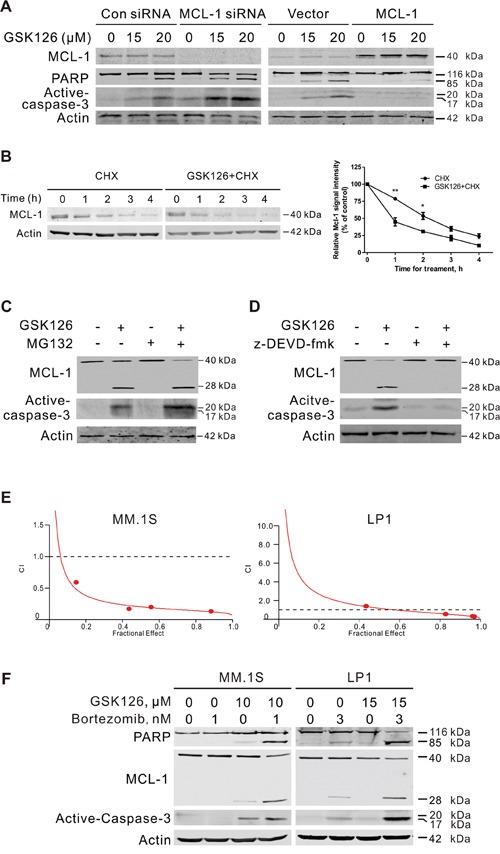
MCL-1 is critical for GSK126-induced apoptosis and involved in synergistic antitumor effect between GSK126 and bortezomib **A**. MM.1S cells were transfected with MCL-1 expression plasmid or siRNA for 24 h, then treated with GSK126 at indicated concentrations for another 24 h. The whole cell lysates were subjected to immunoblotting with MCL-1, PARP and active-caspased-3 antibodies, respectively. **B**. MM.1S cells were treated with 25 μM GSK126 for 12 h, followed by treatment with cycloheximide (CHX, 100 μM) for another 0~4 h. Whole cell lysates were extracted for immunoblotting with anti-MCL-1 (left). Triplicate experiments were performed for statistical analysis (right). Quantification of the signal intensity was done in the Image Studio Lite software (LI-COR, Inc.). *, *P* < 0.05; **, *P* < 0.01, Student's *t* test. **C**. MM.1S cells preincubated with MG132 (50 nM) for 1 h and then were treated with GSK126 (25 μM) for 12 h. The degradation of MCL-1 was analyzed by immunoblotting. **D**. Immunoblotting analysis was performed for the active-caspase-3 and cleavage of MCL-1 in MM.1S cells treated with z-DEVD-fmk (20 μM, 1 h) and following GSK126 (25 μM, 24 h). **E**. MM.1S and LP1 cells were treated with a serial constant-ratio combining GSK126 and bortezomib for 72 h. The cell viability-based synergistic effect of GSK126 and bortezomib was analyzed according to approach described by Chou and Talalay. The combination index (CI) smaller than 1 indicates a synergetic effect. **F**. MM.1S and LP1 cells were treated with GSK126 and bortezomib alone or in combination for 24 h and cell lysates were subjected to immunoblotting with anti-active caspase-3, anti-MCL-1 and anti-PARP.

We next examined the importance of MCL-1 in the GSK126-mediated apoptosis of MM cells by forced overexpression of MCL-1. Overexpression of MCL-1 in MM.1S led to resistance to GSK126-inducing apoptosis, while silencing MCL-1 by siRNA increased sensitivity of MM.1S cells to GSK126-inducing apoptosis as reflected by increase in specific cleavage of PARP and activated caspase-3 (Figure 5A).

We next explored the mechanism by which GSK126 decreased MCL-1. When treated with protein synthesis inhibitor cycloheximide (CHX) and GSK126, MM.1S cells displayed an increased turnover rate of MCL-1 compared to that treated by CHX alone (Figure 5B). MG132 were further used to determine whether the degradation of MCL-1 induced by GSK126 was proteasome-dependent. As expected, MG132 alone blocked the intrinsic degradation of MCL-1 as revealed by immunoblotting (Figure 5C). However, it did not attenuate the GSK126-induced degradation of MCL-1 (Figure 5C), indicating that the degradation was proteasome-independent.

Because MCL-1 is cleaved by caspase-3 to lose its pro-survival effect in apoptosis [22, 23], we treated MM.1S cells with GSK126 in the presence or absence of z-DEVD-fmk (caspase-3 inhibitor). Blocking activation of caspase-3 by z-DEVD-fmk reversed the cleavage of MCL-1 induced by GSK126 (Figure 5D). Together, these data demonstrated that the cleavage of MCL-1 by active-caspase-3 may contribute to the GSK126-induced apoptosis.

### GSK126 synergizes with bortezomib to induce apoptosis of MM cells

Bortezomib is a reversible proteasome inhibitor that was approved by the United States Food and Drug Administration (FDA) for use in relapsed/refractory MM in 2003 and further approved for frontline therapy in combination with other drugs [24, 25]. We tested the synergistic effect of GSK126 and bortezomib. MM.1S and LP1 cells were treated with a serial of fixed-ratio combinations of GSK126 and bortezomib. After 72 h, cell viability was measured by MTS assay and combination index (CI) was used to quantify the combination effect. Synergism (CI < 1) was observed between the two agents (Figure 5E). Immunoblotting results showed that the combination treatment between GSK126 and bortezomib in MM.1S and LP1 cells induced enhanced apoptosis, reflected by specific cleavage of PARP, caspase-3 activation and MCL-1 decrease (Figure 5F).

### GSK126 eliminates CSCs in MM with the Wnt/β-catenin signaling pathway suppressed

**Figure 6:**
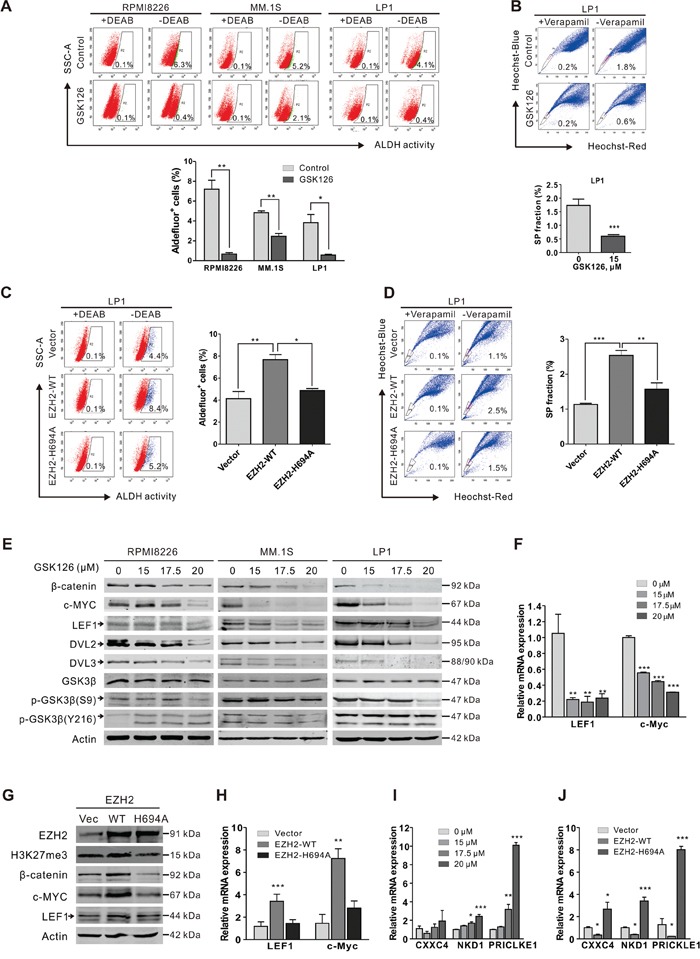
GSK126 eliminates stem-like myeloma cells through blocking of Wnt/β-catenin pathway **A**. RPMI8226, MM.1S and LP1 cells were treated with or without GSK126 (15 μM) for 24 h, and then ALDH^+^ cells were examined using ALDEFLUOR™ Kit (STEMCELL Technologies). DEAB was used as negative control. Representative dot plots (*top*) and statistical analysis (*bottom*) of 3 independent experiments are shown. **B**. LP1 cells were treated with or without GSK126 (15 μM) for 24 h, and the side population (SP) cells were detected by staining with Heochst33342 (5 μg/ml) for 90 min. Veraparmil (50 μM) was used as negative control. **C-D**. After transfected with vector, EZH2-WT and EZH2-H694A plasmids, respectively, LP1 cells were subjected to ALDH (C) and SP (D) assay. **E**. RPMI8226, MM.1S and LP1 cells exposed with indicated concentrations of GSK126. Regulatory proteins in Wnt/β-catenin pathway were analyzed by immunoblotting. **F**. After treatment with different concentrations GSK126 for 24 h, the mRNA expression levels of LEF1, c-Myc in LP1 cells were detected by qRT-PCR. GAPDH was used as a reference gene. **G-H**. After transfected with vector, EZH2-WT and EZH2-H694A plasmids, respectively, LP1 cells were subjected to immunoblotting (G) and qRT-PCR (H) analysis. **I-J**. After LP1 cells were treated with different concentrations GSK126 for 24 h (I) or transfected with vector, EZH2-WT and EZH2-H694A plasmids (J), respectively the mRNA expression levels of CXXC4, NKD1 and PRICKLE1 were detected by qPCR *, *P* < 0.05; **, *P* < 0.01, Student's *t* test. Arrows indicates the specific bands of corresponding proteins.

Because CSCs confer relapse and resistance to chemotherapy in various types of cancers [26], we assessed the ability of GSK126 to kill CSCs in MM. ALDH activity has been used as a stem cell marker for a number of cancer types [27], including MM [28, 29]. We first examined the effects of GSK126 on MM stem cells using ALDH assay. After treatment with 15 μM GSK126 for 24 h, the percentage of ALDH^+^ cells in RPMI8226, MM.1S and LP1 was significantly reduced (Figure 6A). The decrease of CSC subset in MM cells after GSK126 treatment was further confirmed by the results of side population (SP) assay in LP1 cells (Figure 6B). In addition, we transfected the EZH2-WT and EZH2-H694A plasmids into LP1 cells to verify that the HMT activity was indeed required for stemness maintenance in MM. A significant increase in ALDH_+_ and SP fraction cells was observed in the EZH2-WT-overexpressed cells compared to the empty vector control, whereas EZH2-H694A attenuated these phenotypes (Figure 6C). Similar results were obtained by SP evaluation (Figure 6D). These results indicated that loss of HMT activity of EZH2 inhibits the MM stem cell property.

The Wnt/β-catenin pathway is one of the well characterized pathways that are critical for the stemness of CSCs [30], we next tested whether Wnt/β-catenin pathway was engaged in the GSK126-induced elimination of CSCs in MM. Immunoblotting results showed that GSK126 dose-dependently decreased the levels of β-catenin as well as its dependent downstream transcriptional targets (e.g., c-MYC and LEF1) (Figure 6E). qRT-PCR analysis showed that the mRNA expression levels of c-Myc and LEF1 were significantly decreased in the GSK126-treated cells (Figure 6F). Further, ectopic overexpression of EZH2-WT but not the EZH2 H694A-mutant increased the protein levels (Figure 6G) and mRNA levels (Figure 6H) of β-catenin, c-Myc and LEF1. Together, these results indicated that GSK126 treatment or loss of HMT activity of EZH2 down-regulated the activity of Wnt/β-catenin signaling pathway.

We next explored how Wnt/β-catenin signaling pathway was inhibited by GSK126. Analysis of the upstream molecules of β-catenin revealed a decrease in Ser9 phosphorylated GSK3β after GSK126 treatment, but the total and Tyr216 phosphorylated GSK3β were not changed (Figure 6E). However, both DVL2 and DVL3 were decreased (Figure 6E), implying a role of DVL protein in the GSK126-mediated Wnt signaling repression. Indeed, it has been reported that several Wnt signaling pathway inhibitory genes, including CXXC4 (Idax), NKD1 and PRICKLE1, were targets of EZH2 and the repression of these genes by EZH2 promoted the activation of Wnt signaling [31, 32]. CXXC4 [33], NKD1 [34] and PRICKLE1 [35] are all DVL interacting proteins which inhibit the canonical Wnt pathway by their interaction. CXXC4 and NKD1 possibly compete with Axin for the binding to DVL, resulting in the suppression of Wnt pathway [33, 34]; PRICKLE1 binds to DVL and promotes its ubiquitination and proteasomal degradation [35]. As they are EZH2 targets genes, we examined if the expression of these genes was upregulated after GSK126 treatment. As anticipated, the mRNA levels of CXXC4, NKD1 and PRICKLE1 were indeed significantly increased after treatment with GSK126 (Figure 6I). Furthermore, the expression of these DVL antagonists was suppressed by transfection of EZH2-WT plasmid but not EZH2-H694A in MM cells (Figure 6J). Taken together, these results revealed that upregulation of CXXC4, NKD1 and PRICLKE1 and the subsequent inhibition of DVL protein may play a critical role in GSK126-mediated suppression of Wnt/β-catenin signaling pathway.

### GSK126 inhibits xenografted human multiple myeloma growth in nude mice

**Figure 7:**
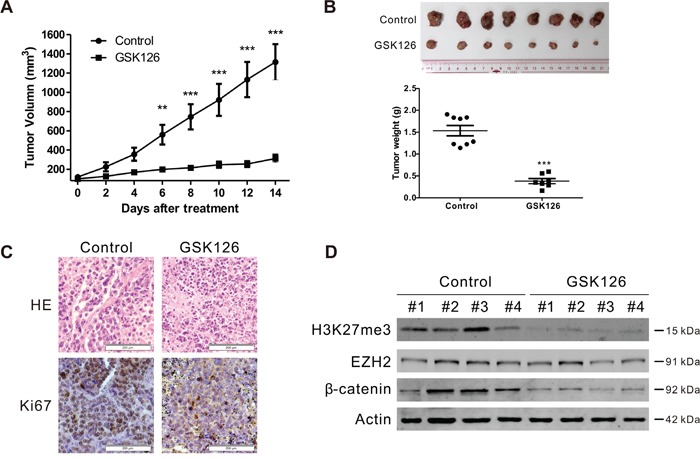
GSK126 abrogates the growth of RPMI8226 cells in subcutaneous xenografts of nude mice **A**. The tumor growth curves were shown. Nude mice with RPMI8226 xenografts were treated with vehicle or GSK126 (200 mg/kg/day, i.p.) for ~14 days. **, *P* < 0.01; ***, *P* < 0.001, Student's *t* test. **B**. Tumors dissected at the endpoints of the experiments were weighed and recorded. Shown are the photograph of the tumors (*top*) and the comparison of the tumor weight of the control and treated groups (*bottom*). ***, *P* < 0.001, Student's *t* test. **C**. Hematoxylin and eosin (H&E)-staining and immunohistochemical analysis were performed for Ki67 in the xenograft tissues. **D**. Lysates of xenograft tissues from 4 representative mice of each group were subjected to immunoblotting with the indicated antibodies.

To evaluate the *in vivo* anti-MM effect of GSK126, RPMI8226 cells were subcutaneously injected into the flanks of nude mice. When the xenografts were palpable (~100 mm^3^), the mice were divided randomly into two groups, receiving vehicle or GSK126, respectively, for 2 weeks; the tumor sizes were measured every other day. The growth of xenografts in mice received GSK126 were significantly delayed compared to the control group (Figure 7A). Likewise, the weights of tumors from the drug-treated group were much lower than that from vehicle group (Figure 7B). Immunohistochemical analysis of Ki67 in the tumor tissue of the two groups showed that a marked decrease of proliferating cells in GSK126-treated mice (Figure 7C), indicating a potent anti-proliferation activity against MM cells of GSK126 *in vivo*. We next detected the inhibitory effect of GSK126 on the methyltransferase activity of EZH2 and the Wnt/β-catenin pathway. Immunoblotting analysis of lysates from 4 tumor tissues each group showed pronounced a decrease in H3K27me3 and β-catenin (Figure 7D).

## DISCUSSION

Targeting the epigenetic alterations may be a promising strategy to abrogate MM. Histone deacetylase (HDAC) inhibitor ACY-1215 has been shown potent activity against MM *in vitro* and *in vivo*, alone or in combination with bortezomib [36]. In the present study, we evaluated the anti-tumor activity of a specific EZH2 inhibitor GSK126, which counters the H3K27me3 increase in MM cells. Our results revealed that GSK126 effectively induced growth inhibition and apoptosis in MM cells as a single agent. When combined with bortezomib, a synergism was observed. Moreover, GSK126 reduced the proportion of ALDH^+^ cells in MM cells, suggesting its ability to eliminate myeloma stem cells. Finally, the activity of GSK126 against MM was confirmed by *in vivo* xenograft mouse model.

EZH2 has been reported to control the proliferation of different normal or tumor cell lines [37–41]. In MM, EZH2 expressions are induced on interleukin 6 (IL-6) stimulation in IL6-dependent cell lines, while constitutively expresses in IL-6-independent cell lines; and the expression of EZH2 is correlative with MM cells’ proliferation, which is abrogated by siRNA treatment [21]. In addition, pharmaceutical inhibition of EZH2 by EPZ6438 blocks the growth of MM cells [42]. In agreement with these results, we found that GSK126 also repressed the growth of several MM cell lines, with IC_50_ ranging from 12.6 μM to 17.4 μM.

Apoptosis in MM cells was induced by GSK126 in a caspase-dependent manner. Mechanistically, GSK126 down-regulated MCL-1 and upregulated BIM which may facilitate triggering the permeabilization of mitochondrial outer membrane, releasing cytochrome c and AIF which then initiated the caspase activation cascade. The endogenous caspase inhibitors XIAP and survivin were also decreased. Among these proteins regulated by GSK126, MCL-1 may be a critical player during the apoptosis, because silencing its expression by siRNA significantly increased the sensitivity of MM.1S cells to GSK126. In contrast, overexpression of MCL-1 by transfection of MCL-1 plasmid caused the resistance of MM.1S to GSK126. Further analysis revealed that MCL-1 was cleaved by caspase-3 during the GSK126-induced apoptosis, which is consistent with previous studies that defined this cleavage and demonstrated its pro-apoptotic function [22, 23]. Of interest, MCL-1 cleavage by caspase-3 is also found and plays a pivotal role in the apoptosis induced by bortezomib [43]. This may in part explained the synergistic effect between GSK126 and bortezomib which we found subsequently.

The reduction of ALDH^+^ cells, a widely accepted parameter reflecting CSCs, in RPMI8226, MM.1S and LP1 cell lines treated with GSK126 indicated a promising application of GSK126 in treatment of MM, as the relapse of MM may be caused by the drug-resistant and self-renewable myeloma stem cells [28, 44, 45]. Previously, EZH2 has been reported to be essential for CSCs of glioblastoma [46, 47] and breast cancer [48]. We found that GSK126 down-regulated the activity of Wnt/β-catenin pathway which is well documented in regulating the self-renewal of normal stem cells and CSCs [49]. The protein levels of β-catenin, its downstream targets c-Myc and LEF1 as well as the upstream components DVL2/3 in MM cells were decreased after exposure to GSK126. In human gastric cancer, EZH2 promotes the activation of Wnt/β-catenin signaling by down-regulating CXXC4 expression [31], and several other Wnt pathway antagonists, including NKD1, PRICKLE1, PPP2R2B, AXIN2 and SFRP5, were concordantly silenced by EZH2 in hepatocellular carcinomas [32]. In the present study, we found that the transcription activities of CXXC4, NKD1 and PRICKLE1 were all significantly upregulated in MM cells following treatment with GSK126, which may help explanation of the inhibition of Wnt/β-catenin pathway.

As the potent anti-tumor effect of GSK126 and other EZH2 inhibitors, preclinical and phase I/II clinical trials have been started evaluating several specific EZH2 inhibitors and promising anti-tumor activity have been obtained [50]. However, resistance to the EZH2 inhibitors has been reported in *in vitro* studies, including mutation of EZH2 [51, 52] and activation of other oncogenic proteins or pathways [53]. In light of these studied, overcoming the EZH2 inhibitor resistance requires development of novel inhibitors or combination strategies with other targeted agents. Combination of bromodomain inhibitor JQ1 with MEK inhibitor PD-901 showed strong activity on killing PRC2-loss-function and NF1 mutant malignant peripheral nerve sheath tumors (MPNSTs) [53], which provides rationality for that combination of GSK126 and bortezomib in our study may be a good way to prevent GSK126 resistance in MM treatment.

In conclusion, we found that the EZH2 inhibitor GSK126 potently inhibits MM cells *in vitro* and *in vivo*. The apoptosis triggered by GSK126 in MM cells is mitochondrial pathway-dependent, in which MCL-1 may play a central role by the cleavage of caspase-3 to generate pro-apoptotic fragments. MCL-1 may also account for the synergism between GSK126 and bortezomib, as both of them induce MM cell apoptosis accompanied by the cleavage of MCL-1. Moreover, the ability of GSK126 to kill the myeloma stem cells augmenting the rationality of applying GSK126 to the treatment of MM as a single agent or in combination with current anti-MM drugs, such as bortezomib.

## MATERIALS AND METHODS

### Chemicals and antibodies

GSK126 (Figure 1A) was purchased from Shanghai Hope Chem Co., Ltd. (Shanghai, China). MG132 was from EMD Millipore Corporation (Billerica, MA). z-DEVD-fmk and bortezomib were from Selleck (Shanghai, China). Antibodies against H3, AIF, BCL-X_L_, MCL-1 (S-19), BIM, GSK3β, BAX, DVL3, Survivin and c-MYC were from Santa Cruz Biotech (Santa Cruz, CA). Antibodies against EZH2, PARP (clone 4C10-5), active-caspase-3, cytochrome c (clone 6H2.B4), XIAP, BCL-2, phospho-GSK3β (Y216), β-catenin were purchased from BD Biosciences (San Jose, CA). Antibodies against H3K27me3, caspase-3, caspase-8, caspase-9, DVL2, and phospho-GSK3β (S9) were from Cell Signaling Technology (Danvers, MA). Antibody against cytochrome c oxidase subunit II (COX II) was from Molecular Probes (Eugene, OR). Antibodies against Axin2 and LEF1 were from Sigma-Aldrich (Shanghai, China). Anti-mouse immunoglobulin G and anti-rabbit immunoglobulin G fluorescent-conjugated secondary antibodies were from LI-COR Biotechnology (Nebraska).

### Cell culture

The MM cell lines MM.1S, U266 and RPMI8226 were purchase from American Type Culture Collection (ATCC, Rockville, MD). LP1, OCI-MY5, JJN3 and OPM2 were kindly provided by Dr. Xinliang Mao, Soochow University, China [54]. These cells were cultured in Iscove's Modified Dulbecco's Media (IMDM, Invitrogen, Shanghai, China) medium supplemented with 10% FBS (Biological Industries, Kibbutz Beit, Haemek, Israel) in a 37°C humidified incubator containing 5% CO_2_.

### Cell viability assay

Cell viability was measured by MTS assay (CellTiter 96® Aqueous Non-Radioactive Cell Proliferation Assay; Promega) as previously described [55, 56]. Briefly, MM cells were seeded into 96-well plates (20,000 cells/well) in triplicate with or without GSK126 for 72 h. MTS supplemented with PMS was added 4 h prior to the end of experiments, and then the absorbance was recorded by a Synergy HT Microplate Reader (Bio Tek) at 490 nm.

### Colony-formation assay

Soft-agar colony-formation assay was performed as previously described [55, 56]. Briefly, MM cells were treated with different concentrations of GSK126 or diluent (DMSO, control) for 24 h, washed with PBS, mixed with drug-free IMDM containing 0.3% agar, and plated into 24-well plates. Colonies were counted after cultured for 10 to 14 days.

### Apoptosis analysis

Apoptosis was measured by Annexin V-fluoresceinisothiocyanate (FITC) and propidium iodide (PI) apoptosis detection kit (Sigma-Aldrich, Shanghai, China) as previously described [57], and analyzed with a BD LSRFortessa flow cytometer (BD Biosciences).

### Mitochondrial transmembrane potential detection

Mitochondrial transmembrane potential (ΔΨm) was measured by the molecular probes MitoTracker Red CMXRos and MitoTracker Green FM (Invitrogen, Shanghai) double-staining. After incubation in 15 μM GSK126 for 24 h, cells were washed with PBS, labeled with the probes at 37°C for 1 h and then subjected to analysis of ΔΨm with flow cytometry [55, 58].

### Immunoblotting analysis

Immunoblotting analysis was performed as previously described [56, 59]. Whole cell lysates prepared in RIPA buffer (1 × PBS, 1% NP-40, 0.5% sodium deoxycholate, 0.1% SDS). For detection of cytochrome c, cytosolic fraction was prepared in digitonin extraction buffer (10 mM PIPES, 0.015% digitonin, 300 mM sucrose, 100 mM NaCl, 3 mM MgCl_2_, 5 mM EDTA, and 1 mM phenylmethylsulfonyl fluoride). Protease and phosphatase inhibitors (1 × protease inhibitor cocktail (Roche), 10 mM β-glycerophosphate, 1 mM sodium orthovanadate, 10 mM NaF, and 1 mM phenylmetnylsulfonyl fluoride) were freshly added to the buffers. The DNA in the lysates was sheared by ultrasonic. Protein concentration was measured by Pierce™ BCA Protein Assay Kit (Thermo Scientific). Equal amounts of protein samples were separated by SDS–PAGE gel electrophoresis and then transferred to nitrocellulose membranes, which were then incubated with the primary antibodies overnight. After incubation with appropriate secondary antibodies, the immunoblots were recorded with the Odyssey infrared imaging system (LI-COR).

### DNA constructs and transfection of plasmids and siRNA duplexes

Human EZH2 expression plasmid pRP[Exp]-mCherry/Puro-EF1A>hEZH2 (EZH2-WT) was constructed by subcloning the full length human EZH2 sequence (NCBI Reference Sequence ID: NM_004456.4) into a pRP[Exp]-CMV>mCherry/Puro vector (cyagen, Guangzhou, China) using Gateway® BP Clonase™ II Enzyme Mix (Invitrogen). The H694A-mutant EZH2 was generated by using the QuickChange Site directed mutagenesis kit (Stratagene). MCL-1 siRNA duplexes and Non-Targeting (mock) siRNA control (Dharmacon RNA Tech., Lafayette, CO.), pCMV5-flag-MCL-1, pRP[Exp]-mCherry/Puro-EF1A>hEZH2, pRP[Exp]-mCherry/Puro-EF1A>hEZH2*H694A and the empty vector plasmids were transfected into MM cells by Nucleofertor™ 2b (LONZA) using Amaxa™ cell line Nucleofector Kit T (LONZA) according to the manufacturer's protocol.

### Quantitative real-time PCR (qPCR)

Total mRNA was extracted by using Trizol reagent (Invitrogen), which then used as templates to generate cDNA with maxima first strand cDNA synthesis kit (Thermo Fisher). Quantitative real-time RT-PCR was performed using SYBR Premix Ex Taq (Perfect Real-time; Takara Bio) according to the manufacturer's instruction in a BIO-RAD CFX96 Real-Time Thermocycler (CFX96, Bio-Rad Laboratories, Hercules, CA). GAPDH was used as the reference gene. The primers used in the study are as follows: CXXC4 forward, 5′-CTCATCAACTGTGGCGTCTG-3′, CXXC4 reversed, 5′-TTAGTTTGCCCTTCATTTCC-3′, NKD1 forward, 5′-TCGCCGGGATAGAAAACTACA-3′, NKD1 reversed, 5′-CAGTTCTGACTTCTGGGCCAC-3′, PRICKLE1 forward, 5′-GACAGTCTCTCCTCTTATCG-3′, PRICLKE1 reversed, 5′-GGATTGAGACTTGGACCTTC-3′, c-Myc forward, 5′-GCCTCAGAGTGCATCGAC-3′, c-Myc reversed, 5′-TCCACAGAAACAACATCG-3′, LEF1 forward, 5′GACGAGATGATCCCCTTCAA-3′, LEF1 reversed, 5′-AGGGCTCCTGAGAGGTTTGT-3′.

### Aldehyde dehydrogenase assay

The ALDH activity of was measured using Aldefluor kit (Stem Cell Technologies, Vancouver, BC, Canada) according to the manufacturer's instruction. Briefly, RPMI8226, MM.1S and LP-1 cells were treated with 15 μM GSK126 for 24 h. 1 × 10^6^ cells were resuspended in 1 ml Assay Buffer with ALDEFLUOR Reagent, separated equally into two tubes, one of which was added with DEAB reagents as negative control. After incubation at 37°C for 1 h, cells were analyzed with BD LRSFortessa flow cytometer [60].

### Side population assay

For side population studies, 1 million cells were incubated with Hoechst 33342 (5 μg/ml) for 90 min at 37°C with a shake every 10 min. Cells incubated with the addition of verapamil (50 μM) were used as a control. Side population cells were analyzed on BD LRSFortessa flow cytometer.

### Nude mouse xenograft model

For *in vivo* experiments, 4- to 6- week-old male nude nu/nu BALB/c mice were purchased from Slac Laboratory Animal Co. (Shanghai, China). Ten million of RPMI8226 suspended in 200 μl PBS were implanted subcutaneously into the flanks of the mice. When the tumors were palpable (~100 mm^3^), mice were randomized into the placebo (n = 8) or experimental groups (n = 8). Administration of GSK126 (200 mg/kg in 20% captisol) and vehicle were achieved by intraperitoneal injection. The mice were euthanized and sacrificed after 2 weeks of treatment; the xenografts were dissected, weighted and fixed. The animal studies was conducted with the approval of the Jinan University Institutional Animal Care and Use Committee.

### Immunohistochemistry

Xenografts dissected from mice at the end of the experiment, were fixed by formalin, embedded in paraffin, and sectioned (4-μm thick). After incubating with anti-Ki67 antibody (Maixin Biol, Fuzhou, China) according to the manufacturer's protocol, the slices were visualized with 0.05% diaminobenzidine and 0.03% H_2_O_2_ in 50 mM Tris–HCl (pH 7.6), and then counterstained with hematoxylin [61].

### Statistics

All experiments were performed at least three times, and data were shown as mean ± standard error of the mean (SEM) unless otherwise indicated. GraphPad Prism 5.0 software (GraphPad Software, San Diego, CA) was used for statistical analysis. Difference between two groups was compared using Two-tailed Student's *t* test and one-way analysis of variance (ANOVA) with *post hoc* intergroup comparison by Tukey's test was used for comparisons among multiple groups. *P* < 0.05 was considered statistically significant.
